# The Prognostic Value of NANO Scale Assessment in IDH-Wild-Type Glioblastoma Patients

**DOI:** 10.3389/fonc.2021.790458

**Published:** 2021-12-02

**Authors:** Johannes Kasper, Tim Wende, Michael Karl Fehrenbach, Florian Wilhelmy, Katja Jähne, Clara Frydrychowicz, Gordian Prasse, Jürgen Meixensberger, Felix Arlt

**Affiliations:** ^1^ Department of Neurosurgery, University Hospital Leipzig, Leipzig, Germany; ^2^ Division of Neuropathology, University Hospital Leipzig, Leipzig, Germany; ^3^ Institute of Neuroradiology, University Hospital Leipzig, Leipzig, Germany

**Keywords:** GBM, glioblastoma, neuro-oncology, neurological performance, NANO scale

## Abstract

**Background:**

IDH-wild-type glioblastoma (GBM) is the most frequent brain-derived malignancy. Despite intense research efforts, it is still associated with a very poor prognosis. Several parameters were identified as prognostic, including general physical performance. In neuro-oncology (NO), special emphasis is put on focal deficits and cognitive (dys-)function. The Neurologic Assessment in Neuro-Oncology (NANO) scale was proposed in order to standardize the assessment of neurological performance in NO. This study evaluated whether NANO scale assessment provides prognostic information in a standardized collective of GBM patients.

**Methods:**

The records of all GBM patients treated between 2014 and 2019 at our facility were retrospectively screened. Inclusion criteria were age over 18 years, at least 3 months postoperative follow-up, and preoperative and postoperative cranial magnetic resonance imaging. The NANO scale was assessed pre- and postoperatively as well as at 3 months follow-up. Univariate and multivariate survival analyses were carried to investigate the prognostic value.

**Results:**

One hundred and thirty-one patients were included. In univariate analysis, poor postoperative neurological performance (HR 1.13, *p* = 0.004), poor neurological performance at 3 months postsurgery (HR 1.37, *p* < 0.001), and neurological deterioration during follow-up (HR 1.38, *p* < 0.001), all assessed *via* the NANO scale, were associated with shorter survival. In multivariate analysis including other prognostic factors such as the extent of resection, adjuvant treatment regimen, or age, NANO scale assessment at 3 months postoperative follow-up was independently associated with survival prediction (HR 1.36, *p* < 0.001). The optimal NANO scale cutoff for patient stratification was 3.5 points.

**Conclusion:**

Neurological performance assessment employing the NANO scale might provide prognostic information in patients suffering from GBM.

## Introduction

IDH-wild-type glioblastoma (GBM) is the most common brain-derived malignancy. Due to its high mitotic activity, neoangiogenesis, and highly infiltrative behavior, it is classified as WHO grade four (1). GBM accounts for 14.5% of all primary brain tumors and is more commonly diagnosed in men. Moreover, the median age at first diagnosis is 65 years and the 12-month survival is poor with around 42.8% (2). Standard therapy includes maximum safe resection and adjuvant radiochemotherapy up to 60.0 Gy with concomitant temozolomide, followed by 6 cycles of temozolomide alone (3, 4). Several parameters were identified as influential on patient survival, including tumor location (5), extent of resection (6), age at date of diagnosis (7), O6-methylguanine-DNA methyltransferase (MGMT) promoter methylation (8), and clinical performance (4). Here, the Karnofsky Performance Score (KPS) is commonly used to assess the overall physical status as well as to monitor possible tumor progression *via* a decrease of clinical performance (9, 10). Moreover, poor or worsened overall neurological performance (11–14) and isolated motor or language deficits might be associated with decreased overall survival (15, 16). In order to address this rising evidence and to standardize the evaluation of neurological performance, the Neurologic Assessment in Neuro-Oncology (NANO) scale was created (17). It was shown to predict overall survival in GBM patients more precisely than comparable performance scales (18, 19). However, previous works are limited by inconsistent therapy regimen within the investigated patient cohorts. Hence, this study was designed to evaluate the independent, prognostic value of neurological performance assessed *via* the NANO scale at different points of follow-up when the abovementioned clinical and radiological factors are considered within a standardized GBM patient collective.

## Methods

### Patient Selection and Treatment

Data collection and analysis were approved by the Ethical Committee of the Medical Faculty, University of Leipzig (No. 144/08-ek). The database of Leipzig University Hospital was searched for all patients with new diagnosis of IDH-wild-type glioblastoma between 2014 and 2019. Inclusion criteria were age over 18 years, at least 3 months postoperative follow-up, and preoperative and postoperative cranial magnetic resonance imaging within 72 h after surgery. Due to the selection criteria, patients with an overall survival less than 3 months or further therapy at another facility were excluded. All cases were discussed in a weekly, interdisciplinary tumor board and therapy regimen was determined based on current EANO guidelines for glioma therapy (20, 21).

### Clinical, Pathological, and Radiological Assessment

Medical records were analyzed for age at date of diagnosis, sex, and adjuvant therapy regimen. The date of diagnosis was set as the date of surgery after neuropathological proof of glioblastoma. Histopathological diagnosis and immunohistochemical status were extracted from neuropathology reports. IDH-mutation status and MGMT promoter methylation of all GBM samples were determined using immunohistochemistry and pyrosequencing or nucleic acid amplification followed by pyrosequencing. According to Quillien et al., the MGMT promoter methylation status was dichotomized into positive (≥12%), negative (<12%), or unknown (22).

Overall survival (OS) was defined as the time between the date of neurosurgery and the date of death. The date of death, if not provided by our hospital database, was collected from the Leipzig Cancer Registry. Dates were assessed on May 31, 2021. If death did not occur by then or if patients were lost to follow-up, the date of last contact to our department was integrated into statistical analysis as censored value.

Tumor location and extent of resection (EOR) were retrospectively determined revising perioperative MRI T1 sequences with and without contrast. Volumetric assessment was manually carried out employing the iPlan Cranial software (version 3.0.5, Brainlab AG, Munich, Germany). If a needle biopsy was performed, EOR was set as 0%.

### Assessment of Neurological Performance Status

All patients appointed to our department are examined based on a standardized procedure including general symptoms, cranial nerve status, sensorimotor deficits, and other focal symptoms, such as aphasia or behavior. Findings are routinely documented within a physician report template. The NANO scale was then retrospectively assessed from physician reports at the time of hospital admission, at the time of discharge, and 3 months postsurgery employing the NANO scale as proposed by Nayak et al. (17). Gait, strength, ataxia of upper extremities, sensation, visual fields, facial strength, language, level of consciousness, and behavior sum up to a maximum of 23 points. High-scale values represent impaired neurological performances. NANO scale changes were calculated by subtracting preoperative scale values from postoperative values (NANO difference 1 or time point 1) or postoperative values from values assessed at 3 months postoperative follow-up (NANO difference 2 or time point 2). NANO scale differences below or equal to 0 represent a stable or improved neurological performance and vice versa. In case of missing data, the corresponding neurological deficit was defined as absent and set as 0 points within NANO scale calculation.

### Statistical Analysis

Statistical analysis was carried out using SPSS statistics software version 24.0.0.2 (IBM, Armonk, NY, USA). First, the assessed parameters were applied as continuous variables and analyzed *via* univariate Cox regression. Time-dependent receiver operator characteristic (ROC) analysis was then performed for NANO scale values with *p*-values below 0.2 from Cox regression, and the optimal cutoff point was defined as the value that maximizes the Youden’s index (parameter value for which sensitivity + specificity − 1 is maximal). After NANO scale values were dichotomized according to cutoff values, a second univariate analysis was carried out employing the Kaplan–Meier estimate. Finally, all continuous variables with *p*-values below 0.2 in univariate Cox regression were utilized for a multivariate analysis *via* proportional hazard Cox regression in order to investigate independent statistical relevance. Non-parametric parameters were compared with Mann–Whitney *U* test.

Survival rates from Kaplan–Meier analysis are given with standard deviation, and statistical significance was calculated *via* log-rank testing. Hazard ratios (HR) are provided with 95% confidence intervals (95 CI). *p*-values below 0.05 were considered statistically significant.

## Results

### Patient Cohort

Baseline data are presented in **Table 1**. Within the study period, 227 patients were newly diagnosed with IDH-wild-type GBM and 131 met the inclusion criteria for the study (a flowchart is shown in **Supplementary Figure 1**). Concerning average age and sex ratio, the cohort is comparable to larger studies (2). The average preoperative NANO scale value was 3.3 ± 2.5 and slightly increased up to 3.8 ± 2.7 at 3 months postsurgery, but statistical significance was not reached (*p* = 0.09 by Mann–Whitney *U* test).

**Table 1:** Baseline data.

No. of patients		131
Sex	Male	88 (67.2)
	Female	43 (32.8)
Average age (years)		65.8 ± 10.2
Tumor location	Frontal	32 (24.4)
	Temporal	37 (28.2)
	Parietal	26 (19.8)
	Occipital	8 (6.0)
	Multilocular	27 (20.6)
	Brainstem	1 (0.8)
Average extent of resection (%)		81.5 ± 29.8
MGMT status	Positive	60 (45.8)
	Negative	67 (51.1)
	Unknown	4 (3.1)
Average NANO	Preoperative	3.3 ± 2.5
	Postoperative	3.6 ± 2.6
	At 3 months postsurgery	3.8 ± 2.7
Adjuvant therapy	RCx	111 (84.7)
	Rx	17 (13.0)
	w/o	3 (2.3)
12-month survival (%)		63.9 ± 4.4

Averages are presented with standard deviation. Percentages of absolute counts are shown in brackets.

MGMT, O6-methylguanine-DNA methyltransferase; NANO, Neurological Assessment in Neuro-Oncology; RCx, radiochemotherapy; Rx, radiotherapy.

### NANO Scale Assessment and Overall Survival (Univariate and Multivariate Analyses)

In univariate Cox regression (see **Supplementary Table 1**), low patient age (HR 1.03, *p* = 0.028), high extent of resection (HR 0.87, *p* < 0.001), adjuvant radiochemotherapy (HR 0.27, *p* < 0.001), positive MGMT promoter methylation status (HR 0.46, *p* = 0.002), and tumor location (HR 0.42, *p* = 0.001) were significantly associated with prolonged survival. Also, a low postoperative NANO scale value (HR 1.13, *p* = 0.004), low NANO scale values at 3 months postsurgery (HR 1.37, *p* < 0.001), and the difference of NANO scale values after 3 months postoperative follow-up and postoperatively (NANO time point 2, HR 1.38, *p* < 0.001) had a significant influence on overall survival. Preoperative NANO values and the difference of post- and preoperative NANO scale values were not associated with patient survival.

**Figure 1:**
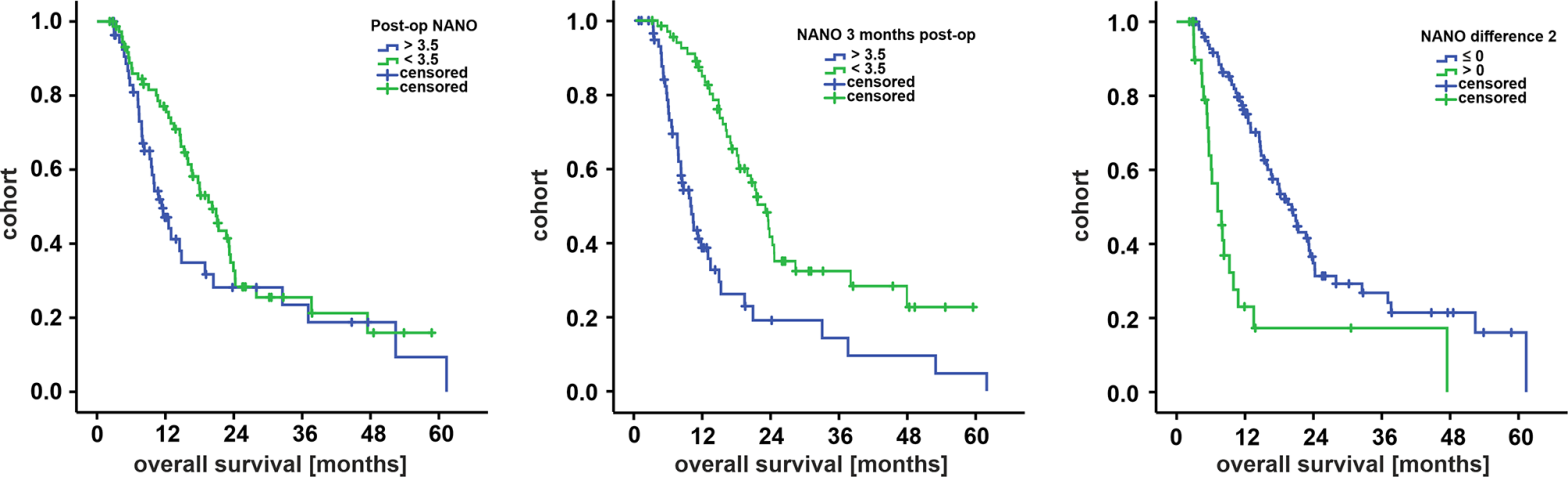
Survival curves for subcohorts by Kaplan–Meier analysis for postoperative NANO scale (left), NANO scale at 3 months follow-up (middle), and NANO scale difference of postoperative values and values at 3 months follow-up (right). Cutoffs for the first two diagrams were determined *via* ROC analysis. NANO difference 2 was dichotomized in stable/increased (≤0) or worsened (>0) neurological performance at 3 months follow-up compared with postoperative values. NANO, Neurologic Assessment in Neuro-Oncology.

A further analysis *via* ROC and Youden’s index calculation revealed 3.5 scale points as the optimal cutoff for both postoperative NANO scale values (AUC 0.706) and NANO scale values at 3 months postoperative follow-up (AUC 0.827). For NANO time point 2, the cutoff was set at 0, with values ≤0 representing stable or increased neurological performance and values >0 representing decreased neurological performance, respectively. Corresponding data were hence dichotomized and employed into Kaplan–Meier analysis, presented in **Figure 1**. Here, patients with NANO scale values below 3.5 points at 3 months postoperative follow-up (12-month survival 85.0 ± 4.4% *vs*. 38.7 ± 6.9%, *p* < 0.001) as well as patients with stable or increased neurological performance (12-month survival 76.2 ± 4.4% *vs*. 23.1 ± 8.6%, *p* < 0.001) had a significantly prolonged overall survival. Postoperative NANO scale assessment was associated with prolonged survival but did not reach significance (12-month survival 77.1 ± 5.0% *vs*. 47.1 ± 7.3%, *p* = 0.056).

Finally, a multivariate Cox regression (**Table 2**) revealed that the extent of resection, adjuvant therapy regimen, MGMT promoter methylation status, and NANO scale assessment at 3 months postoperative follow-up (HR 1.36, *p* < 0.001) were independently associated with increased overall survival.

**Table 2:** Multivariate Cox regression.

	HR	95 CI	*p*-value
Age	1.0	0.97–1.02	0.87
Extent of resection	0.91	0.88–0.99	*0.03*
Location	0.61	0.33–1.12	0.11
Adjuvant therapy	0.45	0.24–0.85	*0.01*
MGMT status	0.54	0.33–0.88	*0.01*
NANO preoperative	1.0	0.89–1.09	0.81
NANO postoperative	0.91	0.78–1.05	0.19
NANO at 3 months	1.36	1.19–1.57	*<0.001*
NANO difference 2^a^	1.39	0.67–2.91	0.38

95 CI, 95% confidence interval; MGMT, O6-methylguanine-DNA methyltransferase; NANO, Neurological Assessment in Neuro-Oncology.

a Difference of NANO scale values at 3 months follow-up and postoperatively. Statistical significance is emphasized in italicized values.

## Discussion

In this retrospective study with 131 IDH-wild-type glioblastoma patients, neurological performance, assessed using the NANO scale, was significantly associated with overall survival in univariate analysis (postoperative, at 3 months postoperative follow-up) and in multivariate survival prediction (at 3 months postoperative follow-up). The optimal NANO scale cutoff for cohort stratification in our series was defined with ROC analysis at 3.5 points. Moreover, patients with NANO scale progression (worsened neurological performance) at 3 months postsurgery suffered from significant shorter OS compared with the corresponding subgroup. It is important to note that due to inclusion criteria (only patients with more than 3 months postoperative follow-up), our cohort mainly consists of “good performers,” reflected by prolonged survival when compared with larger studies. The issue is discussed in detail in the succeeding paragraphs.

The influence of general fitness on the prognosis of cancer patients is universally accepted. KPS and the Eastern Co-operative Oncology Group score/WHO performance scale are universally employed to evaluate the general functional status and are recognized for cancer treatment stratification, including glioma treatment guidelines (21, 23). Commonly, cancer-associated cachexia, chemotherapy toxicity and cancer-associated organ dysfunction are the main causes for an impaired general status. The toxicity of temozolomide is relatively low when compared with other anticancer drugs with thrombocytopenia, neutropenia, fatigue, nausea, and vomiting as the main adverse effects (24, 25). Concerning patients suffering from high-grade glioma on the other hand, neurological deterioration, including focal deficits and cognitive impairment, is pivotal for general performance (26). A decreased preoperative (27, 28) or postoperative (12) neurological performance was shown to be associated with poor prognosis in GBM patients; especially, (newly acquired) aphasia and motoric deficits were found to impair overall survival (15, 29). Moreover, a postoperative decrease of neurological performance has been shown to abrogate the beneficial survival effects gained through an increased extent of GBM resection (11, 13).

All the abovementioned studies employed a non-standardized evaluation of neurological deficits, so the NANO scale was created to objectify clinical assessment in neuro-oncology (17). For glioblastoma patients and in compliance with our results, it was previously shown that NANO scale assessment is significantly associated with patient survival in multivariate survival prediction (19) and might predict overall survival or tumor recurrence more specifically when compared with KPS or ECOG (18). However, there are several limitations to both studies. First, Ung et al. did not evaluate additional clinical and radiological parameters such as the extent of resection, adjuvant radiochemotherapy regimen, or patient age. A NANO scale cutoff is not provided. Second, Lee et al. included patients outside the Stupp regimen defining the present first-line therapy algorithm for glioblastoma. The calculated NANO scale cutoff was 7 points, probably due to an overall reduced neurological performance at initial screening when compared with our data (7.3 ± 3.8 *vs*. 3.3 ± 2.5 in our cohort). Last, both groups did not screen for IDH-mutations and the number of eligible patients did not exceed 80 in either study. In comparison, our data are derived from a larger, homogeneous collective, screened for clinical, radiological, and molecular parameters that are considered essential by current glioma therapy guidelines and classification of CNS tumors (1, 21). This allowed a more coherent interpretation of results and might represent a more reliable database for further projects evaluating the feasibility and prognostic value of neurological performance assessment *via* the NANO scale.

Our study is limited by well-known factors inherent to all retrospective analyses. Selection bias cannot be fully ruled out, especially as all patients with less than 3 months postoperative follow-up are excluded by selection criteria. The time frame was set based on the clinical routine at our center and to allow monitoring neurological performance *via* NANO scale assessment. After receiving the histopathological diagnosis of GBM, patients are directly admitted to adjuvant therapy, followed by 4 to 6 weeks of neurorehabilitation, or vice versa. Hence, the first readmission appointment to our outpatient clinic for initiating maintenance chemotherapy is averagely set 3 months after surgery. Naturally, patients with an extreme short survival period or the wish for palliative care are not recognized by the presented data (55 patients within the study period, see **Supplementary Figure 1**), explaining the prolonged 12-month survival rate shown in **Table 1** when compared with epidemiological analyses (2). This also might have led to a false-negative non-significance of NANO time point 1 (difference of postoperative and preoperative NANO scale values) as patients with an initial extremely poor clinical performance were likely to be ruled out by the study design. Neuro-oncologists should be aware of this especially vulnerable group of patients that is commonly ruled out in projects evaluating new treatment options.

## Conclusion

Monitoring neurological performance *via* the NANO scale might provide prognostic information independently from other well-established clinical, radiological, or pathological factors. Special attention should be paid when worsened neurological performance occurs at the first outpatient appointment after radiochemotherapy and neurorehabilitation. Prospective and multicenter data are needed to further investigate NANO scale assessment in glioblastoma patients, also including a comparison to other performance scales such as KPS or ECOG.

## Data Availability Statement

The raw data supporting the conclusions of this article will be made available by the authors, without undue reservation.

## Ethics Statement

The studies involving human participants were reviewed and approved by the Ethical Committee of the Medical Faculty, University of Leipzig. Written informed consent for participation was not required for this study in accordance with the national legislation and the institutional requirements.

## Author Contributions

Conceptualization: FA, JK, and JM. Data curation: JK, GP, and CF. Formal analysis: JK, MF, TW, and FW. Methodology: JK, MF, TW, and FW. Project administration: JM and FA. Software: GP. Supervision: KJ, FA, and JM. Validation: JM, FA, and KJ. Writing—original draft: JK. Writing—review and editing: all authors. All authors contributed to the article and approved the submitted version.

## Conflict of Interest

The authors declare that the research was conducted in the absence of any commercial or financial relationships that could be construed as a potential conflict of interest.

## Publisher’s Note

All claims expressed in this article are solely those of the authors and do not necessarily represent those of their affiliated organizations, or those of the publisher, the editors and the reviewers. Any product that may be evaluated in this article, or claim that may be made by its manufacturer, is not guaranteed or endorsed by the publisher.

## Abbreviations

95 CI, 95% confidence interval; AUC, area under the curve; EOR, extent of resection; GBM, IDH-wild-type glioblastoma; HR, hazard ratio; MGMT, O6-methylguanine-DNA methyltransferase; NANO, Neurological Assessment in Neuro-Oncology; NO, neuro-oncology; RCx, radiochemotherapy; Rx, radiotherapy; ROC, receiver operator characteristic.

## References

[1] Louis DN Perry A Reifenberger G von Deimling A Figarella-Branger D Cavenee WK. The 2016 World Health Organization Classification of Tumors of the Central Nervous System: A Summary. Acta Neuropathol (2016) 131:803–20. doi: 10.1007/s00401-016-1545-1 27157931

[2] Ostrom QT Patil N Cioffi G Waite K Kruchko C Barnholtz-Sloan JS. CBTRUS Statistical Report: Primary Brain and Other Central Nervous System Tumors Diagnosed in the United States in 2013–2017. Neuro-Oncology (2020) 22:iv1–iv96. doi: 10.1093/neuonc/noaa200 33123732 PMC7596247

[3] Stupp R Mason WP van den Bent MJ Weller M Fisher B Taphoorn MJ. Radiotherapy Plus Concomitant and Adjuvant Temozolomide for Glioblastoma. N Engl J Med (2005) 352:987–96. doi: 10.1056/NEJMoa043330 15758009

[4] Weller M van den Bent M Preusser M Le Rhun E Tonn JC Minniti G. EANO Guidelines on the Diagnosis and Treatment of Diffuse Gliomas of Adulthood. Nat Rev Clin Oncol (2021) 18:170–86. doi: 10.1038/s41571-020-00447-z PMC7904519 33293629

[5] Awad A-W Karsy M Sanai N Spetzler R Zhang Y Xu Y. Impact of Removed Tumor Volume and Location on Patient Outcome in Glioblastoma. J Neurooncol (2017) 135:161–71. doi: 10.1007/s11060-017-2562-1 28685405

[6] Brown TJ Brennan MC Li M Church EW Brandmeir NJ Rakszawski KL. Association of the Extent of Resection With Survival in Glioblastoma: A Systematic Review and Meta-Analysis. JAMA Oncol (2016) 2:1460–9. doi: 10.1001/jamaoncol.2016.1373 PMC6438173 27310651

[7] Okada M Miyake K Tamiya T. Glioblastoma Treatment in the Elderly. Neurol Medico-Chirurgica (2017) 57:667–76. doi: 10.2176/nmc.ra.2017-0009 PMC5735230 29081442

[8] Hegi ME Diserens A-C Gorlia T Hamou M-F de Tribolet N Weller M. MGMT Gene Silencing and Benefit From Temozolomide in Glioblastoma. N Engl J Med (2005) 352:997–1003. doi: 10.1056/NEJMoa043331 15758010

[9] Chambless LB Kistka HM Parker SL Hassam-Malani L McGirt MJ Thompson RC. The Relative Value of Postoperative Versus Preoperative Karnofsky Performance Scale Scores as a Predictor of Survival After Surgical Resection of Glioblastoma Multiforme. J Neurooncol (2015) 121:359–64. doi: 10.1007/s11060-014-1640-x 25344883

[10] Marina O Suh JH Reddy CA Barnett GH Vogelbaum MA Peereboom DM. Treatment Outcomes for Patients With Glioblastoma Multiforme and a Low Karnofsky Performance Scale Score on Presentation to a Tertiary Care Institution. J Neurosurg (2011) 115:220–9. doi: 10.3171/2011.3.JNS10495 21548745

[11] Dietterle J Wende T Wilhelmy F Eisenlöffel C Jähne K Taubenheim S. The Prognostic Value of Peri-Operative Neurological Performance in Glioblastoma Patients. Acta Neurochir (Wien) (2020) 162:417–25. doi: 10.1007/s00701-019-04136-4 31736002

[12] Bleehen NM Stenning SP. A Medical Research Council Trial of Two Radiotherapy Doses in the Treatment of Grades 3 and 4 Astrocytoma. The Medical Research Council Brain Tumour Working Party. Br J Cancer (1991) 64:769–74. doi: 10.1038/bjc.1991.396 PMC1977696 1654987

[13] Rahman M Abbatematteo J de Leo EK Kubilis PS Vaziri S Bova F. The Effects of New or Worsened Postoperative Neurological Deficits on Survival of Patients With Glioblastoma. J Neurosurg (2017) 127:123–31. doi: 10.3171/2016.7.JNS16396 27689459

[14] Verlut C Mouillet G Magnin E Buffet-Miny J Viennet G Cattin F. Age, Neurological Status MRC Scale, and Postoperative Morbidity Are Prognostic Factors in Patients With Glioblastoma Treated by Chemoradiotherapy. Clin Med Insights Oncol (2016) 10:77–82. doi: 10.4137/CMO.S38474 27559302 PMC4990148

[15] McGirt MJ Mukherjee D Chaichana KL Than KD Weingart JD Quinones-Hinojosa A. Association of Surgically Acquired Motor and Language Deficits on Overall Survival After Resection of Glioblastoma Multiforme. Neurosurgery (2009) 65:463–9; discussion 469-70. doi: 10.1227/01.NEU.0000349763.42238.E9 19687690

[16] Sanai N Mirzadeh Z Berger MS. Functional Outcome After Language Mapping for Glioma Resection. N Engl J Med (2008) 358:18–27. doi: 10.1056/NEJMoa067819 18172171

[17] Nayak L DeAngelis LM Brandes AA Peereboom DM Galanis E Lin NU. The Neurologic Assessment in Neuro-Oncology (NANO) Scale: A Tool to Assess Neurologic Function for Integration Into the Response Assessment in Neuro-Oncology (RANO) Criteria. Neuro-Oncology (2017) 19:625–35. doi: 10.1093/neuonc/nox029 PMC5464449 28453751

[18] Ung TH Ney DE Damek D Rusthoven CG Youssef AS Lillehei KO. The Neurologic Assessment in Neuro-Oncology (NANO) Scale as an Assessment Tool for Survival in Patients With Primary Glioblastoma. Neurosurgery (2019) 84:687–95. doi: 10.1093/neuros/nyy098 29618103

[19] Lee J Park SH Kim YZ. Prognostic Evaluation of Neurological Assessment of the Neuro-Oncology Scale in Glioblastoma Patients. Brain Tumor Res Treat (2018) 6:22–30. doi: 10.14791/btrt.2018.6.e1 29644808 PMC5932296

[20] Weller M van den Bent M Hopkins K Tonn JC Stupp R Falini A. EANO Guideline for the Diagnosis and Treatment of Anaplastic Gliomas and Glioblastoma. Lancet Oncol (2014) 15:e395–403. doi: 10.1016/S1470-2045(14)70011-7 25079102

[21] Weller M van den Bent M Tonn JC Stupp R Preusser M Cohen-Jonathan-Moyal E. European Association for Neuro-Oncology (EANO) Guideline on the Diagnosis and Treatment of Adult Astrocytic and Oligodendroglial Gliomas. Lancet Oncol (2017) 18:e315–29. doi: 10.1016/S1470-2045(17)30194-8 28483413

[22] Quillien V Lavenu A Ducray F Joly M-O Chinot O Fina F. Validation of the High-Performance of Pyrosequencing for Clinical MGMT Testing on a Cohort of Glioblastoma Patients From a Prospective Dedicated Multicentric Trial. Oncotarget (2016) 7:61916–29. doi: 10.18632/oncotarget.11322 PMC5308700 27542245

[23] Wick W Osswald M Wick A Winkler F. Treatment of Glioblastoma in Adults. Ther Adv Neurol Disord (2018) 11:1756286418790452. doi: 10.1177/1756286418790452 30083233 PMC6071154

[24] Bae SH Park M-J Lee MM Kim TM Lee S-H Cho SY. Toxicity Profile of Temozolomide in the Treatment of 300 Malignant Glioma Patients in Korea. J Korean Med Sci (2014) 29:980. doi: 10.3346/jkms.2014.29.7.980 25045231 PMC4101787

[25] Scaringi C de Sanctis V Minniti G Enrici RM. Temozolomide-Related Hematologic Toxicity. Onkologie (2013) 36:2. doi: 10.1159/000353752 23921765

[26] Sizoo EM Braam L Postma TJ Pasman HR Heimans JJ Klein M. Symptoms and Problems in the End-of-Life Phase of High-Grade Glioma Patients. Neuro-Oncology (2010) 12:1162–6. doi: 10.1093/neuonc/nop045 PMC3098016 20511193

[27] Chaichana KL Chaichana KK Olivi A Weingart JD Bennett R Brem H. Surgical Outcomes for Older Patients With Glioblastoma Multiforme: Preoperative Factors Associated With Decreased Survival. Clinical Article. J Neurosurg (2011) 114:587–94. doi: 10.3171/2010.8.JNS1081 PMC4020429 20887095

[28] Liang H-K Wang C-W Tseng H-M Huang C-Y Lan K-H Chen Y-H. Preoperative Prognostic Neurologic Index for Glioblastoma Patients Receiving Tumor Resection. Ann Surg Oncol (2014) 21:3992–8. doi: 10.1245/s10434-014-3793-4 24854491

[29] Chaichana KL Pendleton C Chambless L Camara-Quintana J Nathan JK Hassam-Malani L. Multi-Institutional Validation of a Preoperative Scoring System Which Predicts Survival for Patients With Glioblastoma. J Clin Neurosci (2013) 20:1422–6. doi: 10.1016/j.jocn.2013.02.007 PMC4086640 23928040

