# Supplementary figures and images for: The Prognostic Value of NANO Scale Assessment in IDH-Wild-Type Glioblastoma Patients
Source: Front Oncol. 2021 Dec 2;11:790458. doi: 10.3389/fonc.2021.790458 (PMC8674180; doi:10.3389/fonc.2021.790458)

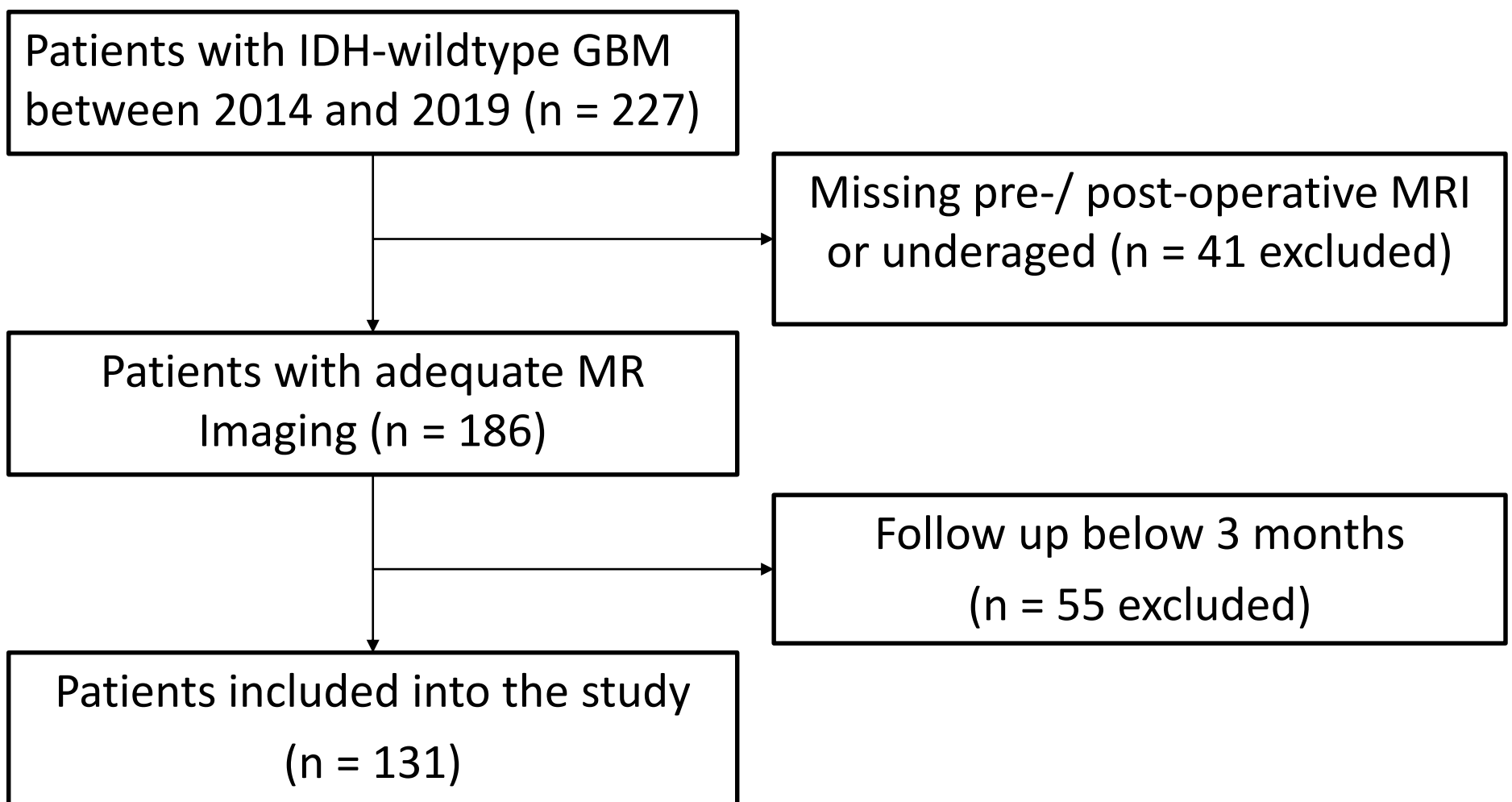

**Suppl. Fig. 1.** Flow Chart.

Supplement: Supplementary file 1 [file DataSheet_1.pdf]
