# Supplementary material for: The Prognostic Value of NANO Scale Assessment in IDH-Wild-Type Glioblastoma Patients
Source: Front Oncol. 2021 Dec 2;11:790458. doi: 10.3389/fonc.2021.790458 (PMC8674180; doi:10.3389/fonc.2021.790458)
Supplement: Supplementary file 2 [file Table_1.docx]

|  | HR | 95CI | p value |
| --- | --- | --- | --- |
| age | 1.03 | 1.00-1.05 | 0.028 |
| extent of resection | 0.87 | 0.89-0.99 | *<0.001* |
| location | 0.42 | 0.25-0.69 | *0.001* |
| adjuvant therapy | 0.27 | 0.16-0.44 | *<0.001* |
| MGMT status | 0.46 | 0.28-0.75 | *0.002* |
| NANO pre-operative | 1.06 | 0.99-1.12 | 0.102 |
| NANO post-operative | 1.13 | 1.04-1.22 | *0.004* |
| NANO at 3 months | 1.37 | 1.25-1.5 | *<0.001* |
| NANO difference 1* | 1.04 | 0.94-1.16 | 0.42 |
| NANO difference 2** | 1.38 | 1.23-1.55 | *<0.001* |

**Suppl. Table 1.** Univariate Cox regression. 95CI: 95% confidence interval; MGMT: O-6-methylguanine-DNA methyltransferase; NANO: neurological assessment in neuro-oncology; *: difference of post-operative and pre-operative NANO scale values; **: difference of NANO scale values at 3 months follow-up and postoperatively.
